# Cost-Effectiveness of Long-Lasting Insecticide-Treated Hammocks in Preventing Malaria in South-Central Vietnam

**DOI:** 10.1371/journal.pone.0058205

**Published:** 2013-03-11

**Authors:** Chantal M. Morel, Ngo Duc Thang, Annette Erhart, Nguyen Xuan Xa, Koen Peeters Grietens, Le Xuan Hung, Le Khan Thuan, Pham Van Ky, Nguyen Manh Hung, Marc Coosemans, Umberto D'Alessandro, Anne Mills

**Affiliations:** 1 LSE Health, London School of Economics & Political Science, London, United Kingdom; 2 National Institute for Malariology, Parasitology and Entomology, Hanoi, Vietnam; 3 Prince Leopold Institute of Tropical Medicine, Antwerp, Belgium; 4 Ninh Thuan Provincial Centre for Malariology, Parasitology and Entomology, Phan Rang, Vietnam; 5 London School of Hygiene & Tropical Medicine, London, United Kingdom; Tulane University School of Public Health and Tropical Medicine, United States of America

## Abstract

**Background:**

Despite much success in reducing the burden of malaria in Vietnam, pockets of malaria persist and eliminating them remains an important development goal. In central Vietnam, insecticide-treated hammocks have recently been introduced to help counter the disease in the highly forested, mountainous areas, where other measures have so far been unsuccessful. This study assesses the cost-effectiveness of using long-lasting insecticide-treated hammocks in this area.

**Methods and Findings:**

This cost-effectiveness study was run alongside a randomized control trial testing the efficacy of the long-lasting insecticide-treated hammocks. Data were collected through an exit survey, a household survey, expenditure records and key informant interviews. The study estimates that under normal (non-trial) conditions the total net societal cost per malaria episode averted in using long-lasting insecticide-treated hammocks in this area was 126 USD. Cost per hammock, including insecticidal netting, sewing, transport, and distribution was found to be approximately 11.76 USD per hammock. Average savings per episode averted were estimated to be $14.60 USD for the health system and 14.37 USD for households (including both direct and indirect cost savings). The study estimates that the annual financial outlay required of government to implement this type of programme to be 3.40 USD per person covered per year.

**Conclusion:**

The study finds that the use of a hammock intervention could represent good value for money to help prevent malaria in more remote areas, where traditional control measures such as insecticide-treated bednets and indoor residual spraying are insufficient or inappropriate to control malaria. However, the life span of the hammock–the number of years over which it effectively deters mosquitoes–has a significant impact on the cost-effectiveness of the intervention and study results should be interpreted in light of the evidence on effectiveness gathered in the years to come.

## Introduction

Despite the overall success of malaria control programmes in Vietnam over the last two decades [1], communities in the south-central part of the country continue to be affected by the disease. These largely forested areas remain attractive biting grounds for highly anthropophilic local vectors [2] that exhibit characteristics (e.g. outdoor, early biting habits) which make them less susceptible to standard interventions such as insecticide-treated mosquito nets (ITNs) or indoor residual spraying [3], [4]. This has led the National Institute of Malariology, Parasitology and Entomology of Vietnam (NIMPE) to seek new methods for controlling malaria in these areas. Between 2004 and 2006 NIMPE, in collaboration with the Institute of Tropical Medicine in Belgium, conducted a large (population 18,646) cluster-based randomized trial to evaluate the efficacy of long-lasting insecticide-treated hammocks (LLIH) for the control of forest malaria in the province of Ninh Thuan, one of most endemic provinces in Central Vietnam. The trial found LLIH to have a 2-fold larger effect on malaria incidence and a 1.6-fold greater reduction in malaria prevalence compared to the control group where routine malaria control measures were applied. This study, conducted in conjunction with the trial, estimates the cost-effectiveness of LLIH in preventing episodes of malaria. It examines the cost-effectiveness of this intervention in a real world (non-trial) context, using a societal perspective.

## Methods

### The Study Area and Population

The highly forested province of Ninh Thuan is located in the south-central part of the country and is largely inhabited by the Ra-glai people who make up 86% of the population [5]. Over the past few decades the Vietnamese government has implemented programs for economic development of the region, implying the relocation of traditional Ra-glai communities to purpose-built villages with basic infrastructure and road access [6], [7]. However, to meet work requirements during the labour intensive rainy season, the Ra-glai continue keep a second home or shelter on the ‘slash and burn’ cultivation fields in the forest [8], [9] where they are exposed to malaria-spreading mosquitoes. Malaria transmission in Ninh Thuan is perennial with peaks in June and October. The study area was made up of 30 villages, comprising 10 communes, over 2 districts (Bac Ai and Ninh Son). These districts see approximately one quarter of all malaria cases occurring overall in the province of Ninh Thuan but counts only 3.5% of the province population.

### Intervention

The distribution of LLIH amongst the Ra-glai community took place as a test arm of a cluster randomized trial in which the control arm continued to be protected by the usual ITNs, while the intervention arm had the ITNs and LLIHs. The new provincial programme also included the use of rapid diagnostic tests (RDTs), and the training of village health workers (VHW) in the use of RDTs and treatment of malaria.

The details of the LLIH trial were reported elsewhere by Thang and colleagues [10]. Seven thousand LLIH were manufactured by the Binh Dinh Textile Company in Quy Nhon province using Olyset® (Sumitomo Chemical Co Ltd, Japan) netting and designed to cover the external layer of the hammock and include a free flap that could be wrapped over the forest-dwelling occupant [11]. Olyset® nets were one of the two LLIN recommended by the WHO Insecticide Evaluation Scheme (WHOPES) at the time of project commencement.

Trial design consisted of the 30 villages (population 18,646) being grouped into 20 clusters (1,000 individuals per cluster) that were randomly allocated to either the intervention or control group (no LLIH) following stratification according to pre-intervention prevalence. The LLIH were distributed to all residents of 10 years of age or more in the intervention clusters in December 2004, attaining coverage of 70% of the intervention population. Coverage was assessed within bi-annual cross-sectional surveys at village level. Participants belonging to the intervention group were asked whether they had received a LLIH and, if so, how they used it (location: village and/or forest; and time: day/evening/night/never) [12]. Incidence of clinical malaria and the prevalence of infection were determined using passive case detection and by bi-annual malariometric surveys.

### Framework of Analysis

The study took a societal perspective, encompassing perspectives of the programme provider, the health system and households. Components of the analysis included programme costs associated with the intervention, the health effects of the intervention (taken from the trial reported in Thang *et al* 2009 [13]), and the resources saved by averting cases of malaria, in terms both of health system resources saved and household resources saved. Household savings included both direct savings (from treatment, transport, etc. averted) and indirect savings (productivity loss averted) associated with the reduction of malaria cases.

### Programme Costs

Costs associated with the LLIH programme were calculated from programme records. As the LLIH were distributed as part of a larger epidemiological research project, assumptions had to be made regarding the time and resource requirements for LLIH distribution only. Based on overall travel times for the 3 mobile teams, it was assumed that distribution of 7,000 LLIH under non-trial conditions would require 10 working days to reach all 15 villages (2 days per village per team), each team being made up of one person from the village and one from province and district authorities, based on procedures for other local distribution programmes. Costs were annualized over the average expected duration of LLIH effectiveness (4 years) and discounted at 3%.

### Health System Costs Averted (Resources Saved in Averting Episodes of Malaria)

#### Diagnosis and treatment costs

Costs associated with malaria diagnosis and treatment were collected from expenditure records at the village, commune health centre (CHC), and hospital levels respectively. Where detailed records were available, treatment costs were built ‘bottom-up’, taking into account the cost of all necessary inputs for diagnosis and treatment including drugs and diagnostics. 53% of uncomplicated cases of malaria were assumed to be infected by Plasmodium falciparum (Pf) as per Thang *et al* [14]. All complicated cases of malaria were assumed to be Pf infections. Uncomplicated cases were assumed to receive Chloroquine (5 tablets per child, 10 tablets per adult) in the case of *P.vivax*, and oral Artesunate (12 tablets per child, 15 tablets per adult) in the case of uncomplicated Pf infection. Complicated Pf patients were assumed to receive quinine injections (3.5 vials per child, 21 vials per adult, over 7 days) and glucose (2×500 ml all patients) as per national treatment guidelines reported by NIMPE staff. In the base case, 30% of the patient population was assumed to be under ten years of age, with an average age of 7 assumed for dosage-weighting as per Morel *et al*. 2008 [15]. The base case scenario also assumed that the RDT Paramax3™ (Zephyr Biomedical) would be used to determine the presence of malaria parasites but confirmed by microscopy at CHC and hospital levels.

#### Labour and capital costs

Each village had one VHW; key information interviews with VHWs were used to estimate the proportion of her/his work time spent on malaria-related activities. As the VHW worked on an *ad hoc* basis no slack time was included in the calculation. VHWs worked from home and in most cases visited ill patients at their homes, which were within walking distance. So, there were assumed to be no other labour-related costs for VHWs. Total CHC labour costs for the 10 CHCs were based on the average CHC staffing of 3 clinically trained members of staff and one assistant. Yearly overheads were assumed to be in line with the Provincial Malaria Station (PMS) expenditure records of 8% of building value. On average CHC staff estimated they spent approximately 18% of their time on malaria-related activities including allowance for slack time. CHC equipment costs consisted of one monocular microscope (lifespan of 15 years) and an average of 3 slide shelves (lifespan of 8 years). The primary purpose of this equipment was to identify malaria infection. CHC buildings were assumed to have a useful life of 30 years. Lifespan of equipment and capital were determined with the help of a NIMPE staff member using aggregate procurement data.

Hospital care was sought primarily for inpatient treatment for severe cases who were referred from the CHC where basic treatment was provided. At hospital level, data on total expenditure only were available, so hospital labour costs per episode were calculated from total inpatient costs using the proportion of inpatient-days attributed to malaria (1,175 malaria inpatient days out of 210,000 inpatient days, or 0.56%) and the number of severe malaria cases seen. Hospital equipment was assumed to be used at a frequency directly associated with the number of inpatient days and therefore apportioned to malaria on this basis. Malaria specific consumables included diagnostic supplies and treatment using quinine injection and glucose drip, based on age-determined quantities. The hospital was assumed to have a useful life of 50 years, hospital equipment 15 years and vehicles (one at the hospital level) of 15 years.

Capital (including equipment) costs at all levels of care were annualized and discounted at 3%. Vietnamese Dong (VND) were converted to US dollars (USD) using the average exchange rate over the period of the epidemiological study, of 15,917 VND per USD and inflated to 2012 USD.

#### Total health system costs

Total diagnosis and treatment costs at each level of care were determined using disease severity, species-specific, and treatment-seeking weights in order to mimic non-trial conditions to the extent possible, drawing largely from Nihn Thuan-based studies [16], [17], [18]. All total yearly cost estimates for malaria-treatment related labour and capital were divided by the average number of yearly episodes of malaria in the study area over the period of the intervention, 2005 and 2006 (742 episodes – almost all of which were uncomplicated). It was assumed that under normal (non-trial) circumstances, 60% of patients would be attended to by the VHW only (uncomplicated cases), 39.76% would attend the CHC only (uncomplicated cases) and 0.24% would attend both CHC and hospital (complicated cases). These estimates are based on a large passive case detection study and epidemiological findings [19], [20].

### Total Household Costs

Data collection methods for household treatment costs associated with malaria, and the estimated costs, are reported in depth elsewhere [21]. As diagnosis and antimalarial treatment were free to patients under the national malaria control programme (i.e. paid by the health system), there were no problems with double counting between household and health system treatment costs. Data on direct and indirect costs of malaria to the household were collected by health workers using exit and household surveys. Patients were asked about all direct costs incurred, including all expenditures associated with seeking treatment and non-medical costs. Indirect costs of malaria to the household were calculated from the estimated productivity loss to the patient’s household due to illness, including reduced production due to the individual’s disease, and indirect costs accrued by the family in caretaking. Estimates of indirect costs from Morel *et al*. 2008 [22] were adjusted to account for more recent findings that suggest slightly fewer complicated cases). Yearly household income was proxied by production (of livestock and agricultural produce) valued at local market prices and converted to average daily household income per person using data on productive days per year, and division of work within the family. Cost-effectiveness calculations were based on findings, reported by Morel *et al* 2008 [23], that uncomplicated and complicated cases lasted 5 and 7 productive person days respectively. These were similar for all ages and, given in-house care provided by healthy adult household members, one-third of a household productive day was lost for each day of ill health in the case of a child episode of malaria and two-thirds in the case of an adult episode.

### Effectiveness

The effects of LLIH were reported by Thang and colleagues [24]. Malaria episodes in intervention and control clusters were monitored using an extensive surveillance system combining bi-annual cross-sectional surveys and passive case detection at the village level. Malaria infection was defined as a positive blood slide with Plasmodium asexual forms, regardless of symptoms and parasite density. Intervention and control groups were comparable for a series of socio-demographic characteristics and for bednet usage, which was over 90% when including untreated bednets. For the base case of the cost-effectiveness analysis the ability of LLIH to reduce the number of malaria episodes was assumed to be maintained over the 4-year life of the LLIH.

### Sensitivity Analyses

Sensitivity analyses were carried out to examine the implications of uncertainty surrounding some of the data and to test results given differing conditions and assumptions. Data values that were considered uncertain or highly variable were replaced with alternate values in order to observe the level of change in the study results. Where parameters were considered highly uncertain, base case estimates were halved, doubled and tripled. The cost of the base hammock (without Olyset® netting), transport of LLIH, and indirect costs per household, were considered potentially highly variable. The proportion of overall cases that were severe was tested at levels of 0, 5%, and 10%. The proportion of cases averted in children under 10 was tested at levels of 50% and 70%. The proportion of cases seen at the village level was tested at levels of 40% and 80%. Life of LLIH was tested at 2 years and 5 years.

## Results

Table 1 shows programme, health system and household costs, unit costs and cost-effectiveness ratios.

**Table 1:** Programme, health system and household costs, unit costs and cost-effectiveness ($2012).

**1. Programme costs**			
*Costs per LLIH*			
Base hammock		4.70	
Olyset netting		5.88	
Sewing of netting		0.76	
Transport		0.42	
Total cost per LLIH		11.76	
*Total annualized, discounted programme costs*		22,139	
**2. Cost per average visit by facility**			
2.1 Village level treatment			
*Recurrent costs*	Cost per year		Cost per episode seen at this level of care
Labour	88		5.37
Diagnostic supplies			1.13
Treatment			0.33
		total	6.83
2.2 CHC level treatment			
*Capital costs*	Cost per year	Cost per item	Cost per episode seen at this level of care
Building		7385	2.63
Equipment		595	1.69
*Recurrent costs*			
Labour	2687		18.76
Diagnostic supplies	42.44		1.43
Treatment			0.34
Overheads	25.64		0.21
		total	25.06
2.3 Hospital level treatment			
*Capital costs*	Cost per year	Cost per item	Cost per episode seen at this level of care
Building		1,477,140	11.47
Vehicle		31,653	0.53
Equipment		886,285	14.84
*Recurrent costs*			
Fuel	2,028		0.40
Labour	627,785		125.44
Consumables	18,464		5.40
Overheads	221,350		44.24
		total	202.32
2.4 Total health systems cost per episode			
	Treatment seeking probabilities in base episode	Cost	Overall cost per average episode
VHW (uncomplicated)	0.6	6.83	4.10
CHC (uncomplicated)	0.3976	25.04	9.96
Hospital		202.32	
CHC+Hospital (severe)	0.0024	227.36	0.54
		total	14.60
3. Household costs per average episode			
Travel			0.11
Treatment			0.68
Indirect (productivity loss)			13.58
		total	14.37
4. Total costs and cost-effectiveness			
Total annualized, discounted programme costs (including 7000 LLIH)		22,139	
Total household costs per episode		14.37	
Total health systems cost per episode		14.60	
Episodes averted through LLIH per year		143	
Gross programme cost per episode averted		155.18	
Total resource savings achieved per year		4,143	
Total cost for LLIH programme per year (net of all resource savings)		17,996	
Net societal cost per episode averted		126	
Net societal cost per episode averted net of household savings		140	
Net societal cost per episode averted net of all resource savings		155	

### Long Lasting Insecticide-treated Hammocks

The total costs per LLIH distributed (including materials, manufacture and distribution) were estimated to be 11.76 USD, made up mainly of the base hammock and netting costs.

### Effect of LLIH on Malaria

The epidemiological study estimated that an average of 143 cases were averted by the LLIH per year in the study area (286 cases were averted over the 2-year study period) [25].

### Total Health System Costs per Malaria Episode

Total cost per episode treated at the village level was 6.83 USD. One VHW cost the health system 88 USD per year and saw an average of 15 patients per year according to treatment-seeking probabilities and total cases in the study area. VHWs were found to work an average of 8.6 hours in total per month, with 90% of their time associated with (uncomplicated) malaria activities.

The total estimated treatment cost per episode at the CHC was estimated to be 25.06 USD. Each CHC saw an average of 30 malaria patients per year according to treatment-seeking probabilities and overall cases in the study area.

The total estimated treatment cost per (severe) episode at hospital level was 202.32 USD. When weighted by treatment-seeking probabilities, total cost to the health system per episode was $14.60 USD (weighted average).

### Total Household Costs per Episode

Total (direct and indirect) household costs were estimated to be 14.37 USD in the base case. Average cost of travel and treatment, of 0.11 USD and 0.68 USD respectively, meant an average total direct household cost of 0.79 USD per episode [26]. The mean weighted by base case age distribution and disease severity resulted in an average indirect cost per case of 13.58 USD, deriving from uncomplicated and severe episodes of malaria amongst productive members of the household (those between the ages of 10 and 65) representing a loss of 15.96 and 22.35 USD respectively. Uncomplicated and severe episodes amongst non-productive members represented a loss of 7.98 and 11.17 USD respectively.

### Total Net Societal Cost per Episode Averted

Using the base case, the total net societal cost per episode averted with an LLIH programme was estimated to be 126 USD (programme costs minus health system and household resource savings from episodes averted). When household resource saving from episodes averted were excluded from the calculations (representing net cost-effectiveness from a government only perspective), the cost per case averted rose to 140 USD. When resource savings to the health system were also excluded (representing gross programme costs) the cost per case averted rose to 155 USD.

### Sensitivity Analysis

Table 2 summarizes results of the sensitivity analysis. The analysis suggests that the useful life of the LLIH is the most significant factor affecting the overall cost-effectiveness of LLIH. The cost of the base hammock is also a major contributing factor. Household indirect costs, severity of disease, and the discount rate have a modest impact on results. Cost of transport has little effect. Age composition of averted cases and proportion of cases seen at the village level make very little difference to cost-effectiveness results.

**Table 2:** Sensitivity analysis.

	Base case value	Testing value	Cost per case averted using testing value	Deviation from base case cost per episode averted	Justification of use of testing value
Cost per base hammock (USD)	4.70	(×0.5)	94.89	−25%	a. (see bottom of table)
		(×2)	187.78	49%	
		(×3)	249.71	98%	
Cost of transport per net (USD)	0.42	(×0.5)	123.14	−2%	b.
		(×2)	131.27	4%	
		(×3)	136.69	9%	
Indirect cost per household per episode (USD)	13.58	(×0.5)	132.64	5%	c.
		(×2)	112.28	−11%	
		(×3)	98.69	−22%	
Overall % of cases that are severe	0.24%	0%	107.48	0%	d.
		2%	103.73	−3%	
		10%	89.70	−16%	
Cases averted in individuals >10 years of age	70%	50%	108.43	1%	e.
		30%	109.81	3%	
		10%	111.19	4%	
Cases in study area seen at the village level	60%	40%	107.31	0%	f.
		80%	106.8	0%	
Useful life of LLIH (years)	4	2	231.18		g.
		5	82.25	−23%	
Discount rate	3%	0%	98.21	−8%	h.
		5%	113.05	6%	

a./b The LLIH were manufactured specifically for this project. For possible transferability of findings the base case values should therefore be considered point estimates for a variable with potential high variability. Base case estimates were halved, doubled, and tripled.

c Indirect costs of malaria to household (from Morel *et al*. 2008) were calculated from the estimated productivity loss to the patient’s household due to illness. As productivity is a function of weather and other factors, it may vary considerably from year to year.

d Evidence on severity of cases in the base case was drawn from years when extremely few severe episodes were detected, which may represent an exception rather than the norm. Higher levels were used in the sensitivity analysis to represent slightly higher (2%), the estimate used in Morel *et al*. 2008 based on a large passive case detection study^47^, and much higher (10%) estimates.

e The base case assumes LLIH protect patients at random, independently of their age. However, episodes averted through use of LLIH may be age dependent ^47^ given that age groups may stay in the forest for different amounts of time and undertake activities which expose them to varying degrees to the malaria vector. The sensitivity analysis tests for an age-effect in either direction, protection largely in adults and largely in children.

f At the time of study, the VHW programme was just getting underway. Over time it could be expected that more malaria patients will come to understand that the VHW is trained to test for and treat malaria. This may increase the treatment seeking activity at the village level.

g Prior to trial commencement the understanding was that LLIH life span would be between 3 and 5 years; however researchers considered at the end of the trial that it might in fact be closer to 2 years (the actual life span could not be measured given that the effectiveness trial lasted 2 years only).

h As per common practice.

## Discussion

Using the base case, the total net societal cost per episode averted with an LLIH programme was estimated to be $126 (2012 USD). It is difficult to compare this result to the cost-effectiveness of other malaria interventions in this particular setting as so little economic exploration has yet been undertaken. Efficacy studies of LLIH have been carried out in Cambodia [27] and in the Amazon region of Venezuela [28]. However, at the time of writing no published cost-effectiveness studies of LLIH had been identified. Generally, compared to other malaria control measures, such as ITNs and IRS, LLIH appear relatively expensive. For example, in terms of cost per episode averted adjusted to common $2012, ITNs have been found to range from a few dollars [29] in Thailand and Togo, to $12 in Tanzania (provided through a voucher scheme) [30], to $47 in the Gambia [31] (impregnation alone), to $59 in India [32]. Cost per episode averted using IRS ranges from $24 in Mozambique [33] to to $106 in India [34]. (The types of costs included in the studies mentioned are highly variable across studies and thus comparable to this study to varying degrees.).

However, for several reasons these other studies may not be directly comparable to this study nor relevant to the Nihn Thuan area or population. The LLIH programme was investigated in an area in which bednets were already being used in the villages. It is therefore not reasonable to compare the cost-effectiveness of LLIH studied here to cost-effectiveness of average ITN or IRS coverage. In the Ninh Thuan context the attractiveness of using LLIH lies in their ability to avert malaria episodes that have not been prevented by ITNs, arguably harder-to-reach populations. As such it may be considered more acceptable to spend a greater amount of money relative to first-call interventions. Further, given the habits of the local population (e.g. sleeping or resting in the forests) [35], [36] and the exophilic nature of the vector (biting largely outdoors) it is not clear whether alternative interventions such as ITN or IRS would be appropriate at all in protecting this population when they are in the forest [37], [38]. No studies looking at the similar addition of one intervention to another could be found for comparison.

The annual financial outlay required of government to implement an LLIH programme ($3.40 per person covered) should be seen in the light of the magnitude and benefits derived from the overall health subsidy that is provided for Nihn Thuan and the Ra-glai population. Ultimately it is likely that the decision to continue or expand the LLIH programme will be based on vertical equity. Indeed, this is a poor population relative to the rest of the country and malaria is lingering longer in central Vietnam than in other areas. Therefore, arguments made on the basis of equity are likely to further justify the use of LLIH programmes in addition to any made on the grounds of cost-effectiveness. Over the more long term, the use of LLIH should also be considered within the framework of health policy and interior policy more generally given the potential change that state-encouraged migration may bring. For example, within a generation (or even just a few decades) such policies may change the behaviour of the Ra-glai. As noted in a study from Thailand, changes in the climate, ecology, economy, and politics are affecting the activities of forest-dwelling populations, their relationships to the land and to other people–and, as a result, their patterns of exposure to malaria [39]. The cost-effectiveness of an LLIH programme should be re-examined in light of such changes.

As highlighted in the sensitivity analysis, the duration of LLIH effectiveness in detering mosquitoes has a significant impact on their cost-effectiveness. When embarking on the pilot programme, malaria authorities had the understanding that the insecticidal effect of the LLIH, its physical integrity, and the related maintenance would result in a life span of 3 to 5 years (hence the base case point estimate of 4 years. While the trial was not long enough to capture the true life span of the LLIH, at the end of the trial, researchers felt the true life span was closer to 2 years. The sensitivity analysis showed that if indeed the life span of the LLIH were to be closer to 2 years, the resulting cost per episode averted would more than double from the base case, to 272 USD. It was unclear, however, whether the seemingly shorter life span was due to the netting technology itself, the way in which the LLIH was sewn, how it was used, or other reasons. If there are improvements in the limiting factor and a life span of 5 years is achieved, there could be a significant (20%) cost reduction. Evidence on the true life span of LLIH should be used to re-evaluate these cost-effectiveness findings in future.

As forest malaria is a problem in many countries–such as Cambodia, Thailand, India, China, Brazil, to name a few–elements of this study may be pertinent elsewhere. As noted in a 1999 study [40], half of the total area of Thailand is an environment that could be suitable for the transmission of forest-related malaria. And, as in Ninh Thuan, bednets alone have been found to inadequately control malaria in many of the forested areas of South-East Asia, possibly because of the exophilic nature of the main local vector Anopheles dirus [41], and IRS is unsuitable because of the life-style of the inhabitants. However, as emphasized by Goodman & Mills [42], it is risky to generalize from one region to another. Effectiveness may vary if there are differences in epidemiological conditions, demographic factors, immunity levels and drug resistance, education levels, and other local cultural factors (which may affect compliance) [43]. Costs will depend on the scale of the intervention, the price of local inputs, the level of existing infrastructure, and population density [44]. Acceptability of the intervention to the local population, as well as the availability of managerial capacity, will affect both costs and effectiveness [45]. These variations may affect the relative cost-effectiveness of LLIH when considered for other settings. It should also be noted that the construction of the LLIH in this programme may be particularly unrepresentative given the informality of the arrangements. Prices attained under more formal organizational procedures or by another purchaser may vary significantly. A rapid exploration of the price of LLIH was undertaken in parallel to help guide potential future programmes. The official price in 2009 for an Olyset roll (available from 200 m up to 300 metres long) was found to be US$0.98/m^2^ CIF, (each hammock uses a bit over 2 m^2^) [46] with variance by volume. As noted in the sensitivity analysis, if the cost per base hammock in this study actually captured a discount and in fact the price were to be double or triple, the overall cost-per episode averted from a similarly organized LLIH programme would increase by 50 and 100% respectively. Finally, it should be noted that since the study took place treatment for malaria in Ninh Thuan Province with artemisinin-based combination therapy has become the norm (at the time of writing it is Artekin). As treatment with more expensive combination therapies takes place the cost of treatment increases and further strengthens the cost-effectiveness argument for preventive interventions such as LLIH.

### Limitations of the Study

The costing of the LLIH programme assumes an absence of ‘keep-up’ costs (e.g. that no new LLIH are needed, no maintenance required, no usage campaign required, etc. over time) to attain similar health effects. The costs of reimpregnation were not explored in this study, however, anecdotal evidence from the trial suggests that the physical state of the LLIHwere not optimal and that reimpregnation would have been unlikely to achieve the same level of effectiveness as the dissemination of new LLIH. If, however, maintenance is needed to continue the level of coverage and usage of the technology over time, such costs must be taken into consideration. They may be considerable. For example, in a study from Mozambique [47], the economic costs per person covered by an IRS campaign were increased by 39% and 31% respectively when management, and monitoring and surveillance, were needed to keep up effectiveness over time. To achieve similar LLIH results over the longer term may require closer management by programme/provincial staff.

While the costs of research were excluded from the costs presented here, that fact that the effectiveness study was part of a trial may have influenced the true cost and effects of the programme. For example, it may have influenced (increased) the level of staff motivation or general awareness of LLIH, which can have knock-on effects on speed and/or thoroughness of LLIH distribution as well as compliance. It may also have affected the amount of information that the population received about LLIH.

It should be noted that, given that so few deaths occur today due to malaria in Nihn Thuan province, death was not taken as an effectiveness measure within this study. In the case of an increase in the severity of malaria seen in Ninh Thuan (due to growing levels of drug resistance or other factors) the cost-effectiveness of LLIH should be re-examined in light of the new evidence, including relative to interventions intended to tackle severe cases more directly.

The base case assumed that a total of 0.24% of the malaria cases seen in an average Ninh Thuan patient population would be severe and that deaths from malaria would be nil. This low number of severe cases may not hold in the years to come. For example, if resistance to first-line therapy increases, there may be a surge in the number of more severe cases, rendering LLIH more cost-effective.

### Conclusions

Insecticide-treated hammocks may represent value for money when used for protection against malaria amongst hard-to-reach populations. While relative to more traditional control measures the intervention appears expensive, in areas where these measures have proven to be inadequate or inappropriate, culturally-adapted LLIH programmes may be an attractive additional measure. Their use in the forested areas of south-central Vietnam, where ITNs alone are insufficient and IRS is inappropriate, appeared relatively cost-effective.

## References

[1] NIMPE (2008) Annual Report of the National Malaria Control Program in Vietnam: NIMPE. Hanoi, Vietnam.

[2] Erhart A, Thang ND, Hung NQ, Toi lV, Hung LX, et al (2004) Forest malaria in Vietnam: a challenge for control. Am J Trop Med Hyg 70: 110–118. 14993619

[3] Trung HD, Van Bortel W, Sochantha T, Keokenchanh K, Quang NT, et al (2004) Malaria transmission and major malaria vectors in different geographical areas of Southeast Asia. Trop Med Int Health 9: 230–7. 15040560 10.1046/j.1365-3156.2003.01179.x

[4] Trung HD, Bortel WV, Sochantha T, Keokenchanh K, Briët OJ, et al (2005) Behavioural heterogeneity of Anopheles species in ecologically different localities in Southeast Asia: a challenge for vector control. Trop Med Int Health 10: 251–62. 15730510 10.1111/j.1365-3156.2004.01378.x

[5] Erhart A, Thang ND, Ky PV, Tinh TT, Van Overmeir C, et al (2005) Epidemiology of forest malaria in central Vietnam: a large scale cross-sectional survey. Malar J 4: 58 doi:10.1186/1475-2875-4-58. 16336671 10.1186/1475-2875-4-58 PMC1325238

[6] Peeters Grietens K, Xa NX, Erhart A, D’Alessandro U (2010) Low Risk Perception of Contracting Malaria among the Ra-glai Ethnic Minority In South-Central Vietnam. Implications for Forest Malaria Control. Malar J 9: 23. 20089152 10.1186/1475-2875-9-23 PMC2823606

[7] Peeters Grietens K, Nguyen XX (2005) Socio-cultural Study on the Use of Long Lasting Insecticidal Hammocks for Malaria Prevention. ITM Internal report. ITM 2005.

[8] Peeters Grietens K, Xa NX, Erhart A, D’Alessandro U (2010) Low Risk Perception of Contracting Malaria among the Ra-glai Ethnic Minority In South-Central Vietnam. Implications for Forest Malaria Control. Malar J 9: 23. 20089152 10.1186/1475-2875-9-23 PMC2823606

[9] Peeters Grietens K, Nguyen Xuan X, Muela Ribera J, Ngo Duc T, van Bortel W, et al (2012) Social Determinants of Long Lasting Insecticidal Hammock-Use Among the Ra-Glai Ethnic Minority in Vietnam: Implications for Forest Malaria Control. PLoS ONE 7(1): e29991. 22253852 10.1371/journal.pone.0029991 PMC3257264

[10] Thang ND, Erhart A, Speybroeck N, Xa NX, Thanh NN, et al (2009) Long-Lasting Insecticidal Hammocks for Controlling Forest Malaria: A Community-Based Trial in a Rural Area of Central Vietnam. PLoS ONE 4(10): e7369 doi:10.1371/journal.pone.0007369. 19809502 10.1371/journal.pone.0007369 PMC2752990

[11] Thang ND, Erhart A, Speybroeck N, Xa NX, Thanh NN, et al (2009) Long-Lasting Insecticidal Hammocks for Controlling Forest Malaria: A Community-Based Trial in a Rural Area of Central Vietnam. PLoS ONE 4(10): e7369 doi:10.1371/journal.pone.0007369. 19809502 10.1371/journal.pone.0007369 PMC2752990

[12] Thang ND, Erhart A, Speybroeck N, Xa NX, Thanh NN, et al (2009) Long-Lasting Insecticidal Hammocks for Controlling Forest Malaria: A Community- Based Trial in a Rural Area of Central Vietnam. PLoS ONE 4(10): e7369 doi:10.1371/journal.pone.0007369. 19809502 10.1371/journal.pone.0007369 PMC2752990

[13] Thang ND, Erhart A, Speybroeck N, Xa NX, Thanh NN, et al (2009) Long-Lasting Insecticidal Hammocks for Controlling Forest Malaria: A Community- Based Trial in a Rural Area of Central Vietnam. PLoS ONE 4(10): e7369 doi:10.1371/journal.pone.0007369. 19809502 10.1371/journal.pone.0007369 PMC2752990

[14] Thang ND, Erhart A, Speybroeck N, Xa NX, Thanh NN, et al (2009) Long-Lasting Insecticidal Hammocks for Controlling Forest Malaria: A Community- Based Trial in a Rural Area of Central Vietnam. PLoS ONE 4(10): e7369 doi:10.1371/journal.pone.0007369. 19809502 10.1371/journal.pone.0007369 PMC2752990

[15] Morel CM, Thang ND, Xa NX, Hung LX, Thuan LK, et al (2008) The economic burden of malaria on the household in south-central Vietnam. Malar J 7: 166. 18752675 10.1186/1475-2875-7-166 PMC2546429

[16] Morel CM, Thang ND, Xa NX, Hung LX, Thuan LK, et al (2008) The economic burden of malaria on the household in south-central Vietnam. Malar J 7: 166. 18752675 10.1186/1475-2875-7-166 PMC2546429

[17] Thang ND, Erhart A, Speybroeck N, Xa NX, Thanh NN, et al (2009) Long-Lasting Insecticidal Hammocks for Controlling Forest Malaria: A Community- Based Trial in a Rural Area of Central Vietnam. PLoS ONE 4(10): e7369 doi:10.1371/journal.pone.0007369. 19809502 10.1371/journal.pone.0007369 PMC2752990

[18] Thang ND, Erhart A, Speybroeck N, Xa NX, Thanh NN, et al (2009) Long-Lasting Insecticidal Hammocks for Controlling Forest Malaria: A Community- Based Trial in a Rural Area of Central Vietnam. PLoS ONE 4(10): e7369 doi:10.1371/journal.pone.0007369. 19809502 10.1371/journal.pone.0007369 PMC2752990

[19] Thang ND, Erhart A, Xuan HL, Khanh TL, Xuan XN, et al.. (2008) Malaria in Central Vietnam: epidemiological characteristics by cross sectional surveys and passive case detections.

[20] Thang ND, Erhart A, Speybroeck N, Xa NX, Thanh NN, et al (2009) Long-Lasting Insecticidal Hammocks for Controlling Forest Malaria: A Community- Based Trial in a Rural Area of Central Vietnam. PLoS ONE 4(10): e7369 doi:10.1371/journal.pone.0007369. 19809502 10.1371/journal.pone.0007369 PMC2752990

[21] Morel CM, Thang ND, Xa NX, Hung LX, Thuan LK, et al (2008) The economic burden of malaria on the household in south-central Vietnam. Malar J 7: 166. 18752675 10.1186/1475-2875-7-166 PMC2546429

[22] Morel CM, Thang ND, Xa NX, Hung LX, Thuan LK, et al (2008) The economic burden of malaria on the household in south-central Vietnam. Malar J 7: 166. 18752675 10.1186/1475-2875-7-166 PMC2546429

[23] Morel CM, Thang ND, Xa NX, Hung LX, Thuan LK, et al (2008) The economic burden of malaria on the household in south-central Vietnam. Malar J 7: 166. 18752675 10.1186/1475-2875-7-166 PMC2546429

[24] Thang ND, Erhart A, Speybroeck N, Xa NX, Thanh NN, et al (2009) Long-Lasting Insecticidal Hammocks for Controlling Forest Malaria: A Community- Based Trial in a Rural Area of Central Vietnam. PLoS ONE 4(10): e7369 doi:10.1371/journal.pone.0007369. 19809502 10.1371/journal.pone.0007369 PMC2752990

[25] Thang ND, Erhart A, Speybroeck N, Xa NX, Thanh NN, et al (2009) Long-Lasting Insecticidal Hammocks for Controlling Forest Malaria: A Community- Based Trial in a Rural Area of Central Vietnam. PLoS ONE 4(10): e7369 doi:10.1371/journal.pone.0007369. 19809502 10.1371/journal.pone.0007369 PMC2752990

[26] Morel CM, Thang ND, Xa NX, Hung LX, Thuan LK, et al (2008) The economic burden of malaria on the household in south-central Vietnam. Malar J 7: 166. 18752675 10.1186/1475-2875-7-166 PMC2546429

[27] T Sochantha, W Van Bortel, S Savonnaroth, T Marcotty, N Speybroeck, et al.. (2009) Personal protection by long-lasting insecticidal hammocks against the bites of forest malaria vectors. Trop Med Int Health. 15 no 3 336–341. 10.1111/j.1365-3156.2009.02457.x 20070632

[28] Magris M, Rubio-Palis Y, Alexander N et al.. (2007) Community-randomized trial of lambdacylohalothrin-treated hammock nets for malaria control in Yanomani communities in the Amazon region of Venezuela. Trop Med Int Health 12, 392–403. 10.1111/j.1365-3156.2006.01801.x 17313511

[29] Kamolratanakul P, Butraporn P, Prasittisuk M, Prasittisuk C, Indaratna K (2001) Cost-effectiveness and sustainability of lambdadacyhalothrin-treated mosquito nets in comparison to DDT spraying for malaria control in western Thailand. Am J Trop Med Hyg 65: 279–284. 11693869 10.4269/ajtmh.2001.65.279

[30] Mulligan JA, Yukich J, Hanson K (2008) Costs and effects of the Tanzanian national voucher scheme for insecticide-treated nets. Malar J 7: 32. 18279509 10.1186/1475-2875-7-32 PMC2279140

[31] Picard J, Aikins M, Alonso PL, Armstrong Schellenberg JRM, Greenwood BM et al.. (1993) A malaria control trial using insecticide-treated bed nets and targeted chemoprophylaxis in a rural area of The Gambia, West Africa: 8. Cost-effectiveness of bed net impregnation alone or combined with chemoprophylaxis in preventing mortality and morbidity from malaria in Gambian children. Trans R Soc Trop Med Hyg 87, Supp2, 53–57. 10.1016/0035-9203(93)90176-q 8212110

[32] Bhatia MR, Fox-Rushby J, Mills A (2004) Cost-effectiveness of malaria control interventions when malaria mortality is low: insecticide-treated nets versus in-house residual spraying in India. Soc Sci Med 59 525–539. 10.1016/j.socscimed.2003.11.005 15144762

[33] Conteh L, Sharp BL, Streat E, Barreto A, Konar S (2004) The cost and cost-effectiveness of malaria vector control by residual insecticide house-spraying in southern Mozambique: a rural and urban analysis. Trop Med Int Health volume 9 no 1 125–132. 10.1046/j.1365-3156.2003.01150.x 14728616

[34] Bhatia MR, Fox-Rushby J, Mills A (2004) Cost-effectiveness of malaria control interventions when malaria mortality is low: insecticide-treated nets versus in-house residual spraying in India. Soc Sci Med 59 525–539. 10.1016/j.socscimed.2003.11.005 15144762

[35] Peeters Grietens K, Xa NX, Erhart A, D’Alessandro U (2010) Low Risk Perception of Contracting Malaria among the Ra-glai Ethnic Minority In South-Central Vietnam. Implications for Forest Malaria Control. Malar J 9: 23. 20089152 10.1186/1475-2875-9-23 PMC2823606

[36] Peeters Grietens K, Nguyen XX (2005) Socio-cultural Study on the Use of Long Lasting Insecticidal Hammocks for Malaria Prevention. ITM Internal report. ITM.

[37] Trung HD, Van Bortel W, Sochantha T, Keokenchanh K, Quang NT, et al (2004) Malaria transmission and major malaria vectors in different geographical areas of Southeast Asia. Trop Med Int Health 9: 230–7. 15040560 10.1046/j.1365-3156.2003.01179.x

[38] Trung HD, Bortel WV, Sochantha T, Keokenchanh K, Briët OJ, et al (2005) Behavioural heterogeneity of Anopheles species in ecologically different localities in Southeast Asia: a challenge for vector control. Trop Med Int Health 10: 251–62. 15730510 10.1111/j.1365-3156.2004.01378.x

[39] Markwardt R, Sorosjinda-Nunthawarasilp P and Saisang V (2008) Human activities contributing to a malaria outbreak in Thong Pha Phum District, Kanchanaburi, Thailand Southeast Asian J Trop Med Public Health 39 (suppl 1).

[40] Prothero RM (1999) Malaria, forests and people in Southeast Asia. Singapore J Trop Geo 20: 76–85.

[41] Trung HD, Bortel WV, Sochantha T, Keokenchanh K, Briët OJ, et al (2005) Behavioural heterogeneity of Anopheles species in ecologically different localities in Southeast Asia: a challenge for vector control. Trop Med Int Health 10: 251–62. 15730510 10.1111/j.1365-3156.2004.01378.x

[42] Goodman CA, Mill AJ The evidence base on the cost-effectiveness of malaria control measures in Africa. Health Policy Plan 14(4): 301–312. 10787646 10.1093/heapol/14.4.301

[43] Goodman CA, Mill AJ The evidence base on the cost-effectiveness of malaria control measures in Africa. Health Policy Plan 14(4): 301–312. 10787646 10.1093/heapol/14.4.301

[44] Goodman CA, Mill AJ The evidence base on the cost-effectiveness of malaria control measures in Africa. Health Policy Plan 14(4): 301–312. 10787646 10.1093/heapol/14.4.301

[45] Goodman CA, Mill AJ The evidence base on the cost-effectiveness of malaria control measures in Africa. Health Policy Plan 14(4): 301–312. 10787646 10.1093/heapol/14.4.301

[46] Thang ND, Erhart A, Speybroeck N, Xa NX, Thanh NN, et al (2009) Long-Lasting Insecticidal Hammocks for Controlling Forest Malaria: A Community- Based Trial in a Rural Area of Central Vietnam. PLoS ONE 4(10): e7369 doi:10.1371/journal.pone.0007369. 19809502 10.1371/journal.pone.0007369 PMC2752990

[47] Conteh L, Sharp BL, Streat E, Barreto A, Konar S (2004) The cost and cost-effectiveness of malaria vector control by residual insecticide house-spraying in southern Mozambique: a rural and urban analysis. Trop Med Int Health volume 9 no 1 125–132. 10.1046/j.1365-3156.2003.01150.x 14728616

